# Post-Partem Hemorrhage

**Published:** 1876-12

**Authors:** 


Post-Partem Hemorrhage.—We are in receipt of a four-paged pamphlet on the treatment of uterine hemorrhage after delivery, by H. Otis Hyatt, of Kinston, N. C., in which he advises the introduction of a rubber bag, and inflating it with air or water, the latter preferable.

While, in the treatment of hemorrhage after delivery at the full term, we have usually found no difficulty in arresting excessive flow, by introducing the hand into the cavity of the uterus, thereby inducing contraction of the organ and arrest of hemorrhage; yet the plan suggested by Dr. Hyatt is ingenious, and doubtless may be successfully adopted for the purpose. Not only will it afford mechanical pressure by which the bleeding is directly staunched, as well as by promoting contraction; but when cold water is injected into the bag, the refrigeration will certainly add to the styptic influence of the procedure.

Accoucheurs, to make this process available, should always be supplied with India-rubber bags of sizes suitable for abortion and delivery at full term.

